# Topographic Variations of Choroidal Thickness in Healthy Eyes on Swept-Source Optical Coherence Tomography

**DOI:** 10.1167/iovs.61.3.38

**Published:** 2020-03-20

**Authors:** Sara Touhami, Elise Philippakis, Sarah Mrejen, Aude Couturier, Céline Casteran, Priscille Levent, Ramin Tadayoni, Alain Gaudric

**Affiliations:** 1 Ophthalmology Department, Hôpital Lariboisière, Assistance Publique-Hôpitaux de Paris, Université de Paris, Paris, France; 2 Ophthalmology Department, Reference Center in Rare Diseases, DHU Sight Restore, Hôpital Pitié Salpêtrière, Sorbonne Université, Paris, France; 3 Ophthalmology Department, CHNO des Quinze-Vingts, Paris, France

**Keywords:** choroidal thickness, choroidal veins, optical coherence tomography, swept-source, pachychoroid, healthy eyes

## Abstract

**Purpose:**

To assess topographic variations of choroidal thickness (CT) in the fovea and beyond in healthy eyes.

**Methods:**

This cross-sectional study included healthy subjects ≤ 55 years of age with axial lengths (22–26 mm) and refractive error margins (–4D, +4D) in normal ranges. Images were acquired using swept-source optical coherence tomography angiography (OCT-A). Corneal thickness (CT) maps from 12 × 12-mm radial scans and 9 × 9-mm OCT-A B-scans were analyzed.

**Results:**

The study included 64 eyes of 33 subjects (mean age, 37 years). Mean CT was >300 µm in all locations except the nasal outer macula. The subfoveal CT was >395 µm in 30% of cases; in 38.7% of cases, >50% of the CT map was thicker than 395 µm. The mean thickest choroidal point was 395.2 µm (range, 164–548 µm), located superior and temporal to the macula in 72.2% of cases and subfoveally in 1.8% of cases. The CT pattern was symmetrical (58%) or asymmetrical (42%) along a horizontal axis correlating with choroidal vein distribution. Half of the asymmetrical patterns were thicker in the inferior quadrants, with an oblique temporal watershed of venous drainage, and the other half were thicker superiorly. The mean vascularity index was ∼75% regardless of the mean CT.

**Conclusions:**

One-third of healthy eyes of patients younger than age 55 had a thick choroid (>395 µm). In these normal eyes, the thickest choroidal point was not subfoveal, CT symmetry above and below the fovea depended on choroidal vein distribution, and choroidal vascularity index was independent from CT. No patients demonstrated fundus autofluorescence abnormalities, and the choriocapillaris remained visible even in thick choroids. These features could be interesting when differentiating normal versus pathological states.

Since the first measurement of the choroidal thickness (CT) by optical coherence tomography (OCT) in 2008,^1^ there has been a growing interest in assessing the impact of choroidal morphological changes in a variety of chorioretinal disorders.^2^^–^^5^ CT and vascular changes on OCT have been shown to be strong biomarkers that correlate with the axial length (AL)^6^ and visual prognosis in high myopia^7^ or with the pathogenesis^8^^,^^9^ and treatment response in central serous chorioretinopathy (CSC).^10^ The term “pachychoroid” was initially coined to describe eyes with retinal pigment epithelium (RPE) abnormalities associated with choroidal thickening but without any history of subretinal fluid.^11^ This definition subsequently evolved to that of pachychoroid disease, which involved several pathological conditions having in common a thick choroid, pachyvessels, and a thinning of the inner choroid with or without RPE abnormalities.^12^ However, although the CT per se is not considered the most important criterion for defining the pachychoroid disease phenotype,^12^ the threshold of choroidal thickening may be difficult to determine in a single individual or in a group of patients. Many recent publications have studied CT, choroidal vascularity, or vascular topography of the choroid in healthy subjects with variable results.^13^^–^^44^ The use of swept-source OCT (SS-OCT) devices operating at a longer wavelength than spectral domain OCT (SD-OCT) has allowed a more detailed visualization of the choroid. OCT angiography (OCT-A) now provides an additional tool for analyzing the choroidal circulation. The aim of this study was to use a comprehensive methodology, based on the combination of the OCT-A and SS-OCT technologies, to provide a simultaneous analysis of both en face and three-dimensional data on the features of the healthy choroid.

## Methods

### Data Collection

This prospective cross-sectional study was conducted in the Department of Ophthalmology of Lariboisière University Hospital. To avoid the impact of age on CT,^13^^,^^16^ healthy adult subjects 55 years of age or less were included between December 2017 and March 2018. The protocol of the study and the procedures used were approved by the Ethic Committee of the French Society of Ophthalmology (IRB 00008855 Société Française d'Ophtalmologie IRB#1). This research adhered to the tenets of the Declaration of Helsinki. Written informed consent was obtained from all participants.

Normal healthy subjects younger than 55 years with no history of ocular or systemic disorder and with no history of ocular surgery or treatment were included. Refractive error margins were set to the following spherical equivalent (SE) and AL ranges: –4 D < SE < +4 D and 22 mm < AL <26 mm.^15^ All participants underwent a detailed ocular examination including best-corrected visual acuity using a Snellen chart, slit-lamp biomicroscopy, measurement of intraocular pressure (IOP), and autorefractometry (TonoRef II; Nidek, Gamagori, Japan), AL measurement (IOL-Master 700; Carl Zeiss Meditec AG, Jena, Germany), and fundus photographs of the posterior pole, including both color (45°, 5 megapixels) and infrared (82°, 5 megapixels) images (TritonOakland, NJ, USA). All investigations were performed between 1 PM and 3 PM to limit the effects of the circadian rhythm on CT.^45^ Blood pressure was also measured in all cases.

### Image Acquisition

Choroidal images were acquired using the swept-source Topcon DRI OCT Triton with the following features: 1050-µm wavelength, imaging speed of 100,000 A scans per second, axial resolution of 2.3 µm, and transverse resolution of 20 µm. We obtained 12 radial B-scans (12 mm long) and a volume of 512 B-scans using the 9 × 9-mm OCT-A mode, both centered on the fovea. Automatic layer segmentation using Topcon Advanced Boundary Segmentation software was verified by two observers (ST, EP) and manually corrected when necessary by one experienced retina specialist (ST) for each of the 12-mm radial scans to determine choroidal boundaries (inner boundary: Bruch's membrane; outer boundary: choroid–scleral interface [CSI]). Both observers had to agree on the need for manual correction before modifying any of the original segmentations. CT measurements and CT maps were obtained from the 12 × 12-mm radial scans. CT maps derived from the 9 × 9-mm OCT-A scans were based on an automatic segmentation using the manufacturer's software. En face images were obtained from the 9 × 9-mm OCT-A acquisition. The 12-mm B-scans were used for the quantitative analysis of CT values and CT topography, and B-scans derived from the 9 × 9-mm OCT-A acquisition were used to determine the location of the thickest choroidal point (TCP) and comparison with choroidal vessels topography. Autofluorescence (532 nm) images were obtained using Optos (Dunfermline, UK). Subjects with poor OCT image quality were excluded.

### Data Analysis

#### Quantitative Measurements of Choroidal Thick-ness

**Figure 1.**
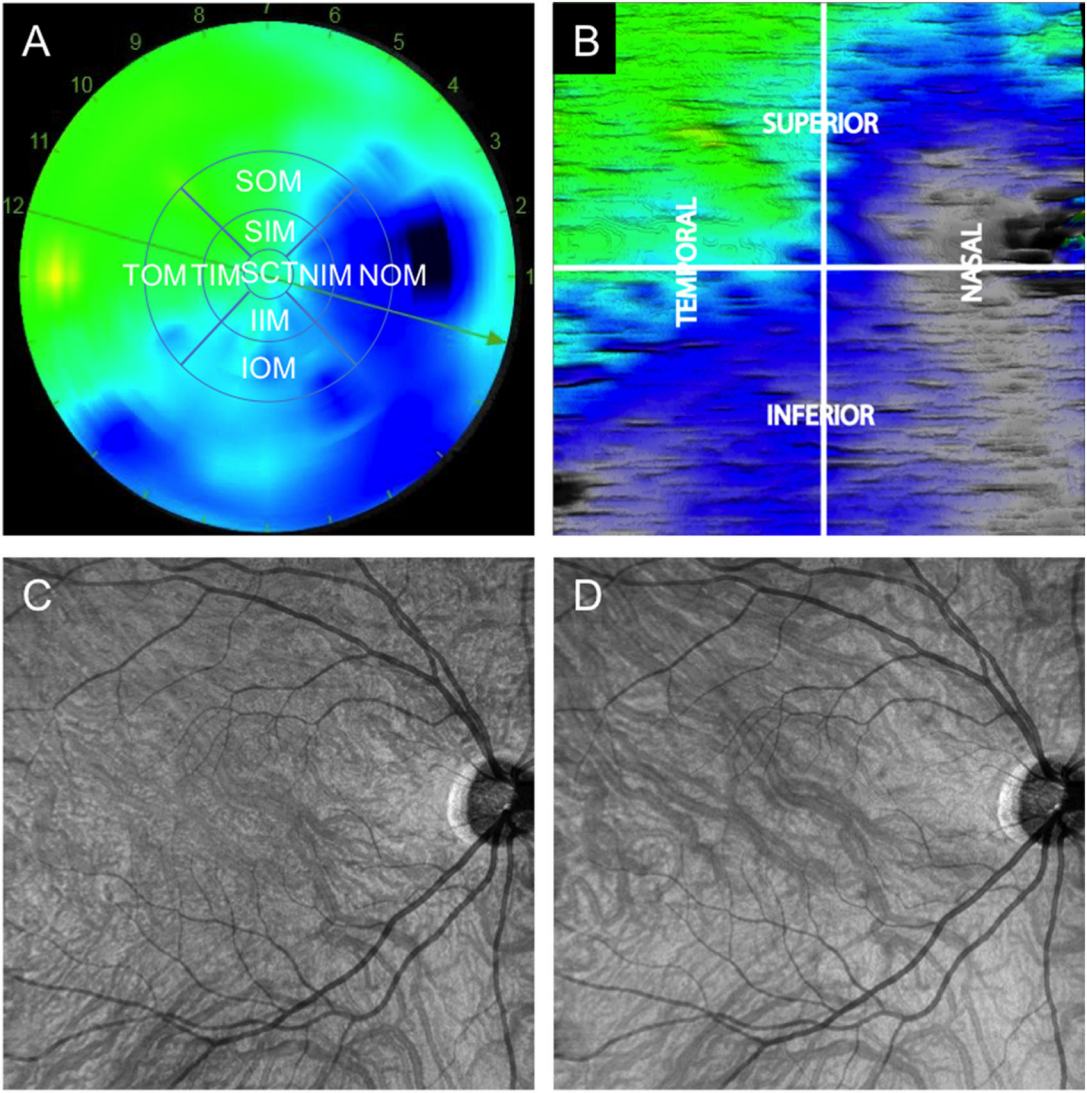
Example of choroidal thickness maps and corresponding en face OCT of the choroid. (**A**) Choroidal thickness (CT) map obtained from 12-mm radial B-scans provided an automatic measurement of the subfoveal CT (SCT) in the central 1-mm-diameter and the mean macular CT in nine subfields based on the ETDRS grid. The inner ring (3-mm radius) included the superior inner macula (SIM), temporal inner macula (TIM), inferior inner macula (IIM), and nasal inner macula (NIM); the outer ring (6-mm radius), the superior outer macula (SOM), temporal outer macula (TOM), inferior outer macula (IOM), and nasal outer macula (NOM). (**B**) Choroidal map obtained from the 9 × 9-mm OCT-A scan shows four quadrants centered on the fovea (superotemporal quadrant, superonasal quadrant, inferotemporal quadrant, and inferonasal quadrant) and allows measuring CT at any point of the map. The thickest choroidal point was determined on this map. (**C**) En face OCT images extracted from the 9 × 9-mm OCT-A scans showing choroidal vessel distribution at 100-µm depth from the retinal pigment epithelium. (**D**) To describe the course of choroidal vessels, we determined that a Bruch's membrane CSI slab covering the entire CT allowed the best visualization of the Haller's layer.

The choroidal map obtained from the 12-mm radial scans provided an automatic measurement of the subfoveal CT (central 1 mm) and the mean CT in nine subfields based on the Early Treatment Diabetic Retinopathy Study (ETDRS) grid (Fig. 1A). The inner ring (3-mm diameter) included the superior inner macula, temporal inner macula, inferior inner macula, and nasal inner macula. The outer ring (6-mm diameter) was comprised of the superior outer macula, temporal outer macula, inferior outer macula, and nasal outer macula (NOM). In addition, an aggregate field was defined as the total macula, corresponding to the mean CT of the nine subfields. The mean and SD and the coefficient of variation (CV) of CT in each macular subfield (= SD/mean) were calculated. The TCP was determined on the 9 × 9-mm OCT-A CT map. The location of the TCP was determined by graphical representation of CT maps using the built-in manufacturer's IMAGEnet 6 software and manually verified for all patients with the caliper by the observer (ST) on the corresponding B-scans. The position of the TCP was recorded on the choroidal thickness map, segmented into four quadrants (Fig. 1B). Furthermore, in order to allow a comparison with the previously published data,^9^^,^^12^ subfoveal CT measurements were also performed using a spectral-domain enhanced depth imaging technology (Spectralis OCT; Heidelberg Engineering, Heidelberg, Germany) in a random sample of 10 eyes from this cohort.

#### Qualitative Analysis of Choroidal Thickness Maps: Patterns and Symmetry

CT maps were obtained from the 12-mm SS-OCT scans according to a color code provided by the IMAGEnet 6 software (red, >400 µm; yellow, 300–400 µm; green, 200–300 µm; blue, <200 µm). To provide an overview of choroidal topography patterns in a healthy population (younger than 55 years of age), the subfoveal CT was not used alone to classify a choroid as thick or thin. The choroid was considered thick when >50% of the map surface was greater than 395 µm (colored in red) and medium-thin in the other cases; measurements were performed using ImageJ software (National Institutes of Health, Bethesda, MD, USA). In the absence of a statistically confirmed value in the existing literature, the threshold of 395 µm was chosen based on a recent publication including patients with a mean age of 47 years, in which a subfoveal thickness > 395 µm (Spectralis) was proposed to define a pathological (CSC) versus non-pathological choroid.^9^ This threshold was used thereafter to define the term “pachychoroid.”^12^ We therefore also investigated the correlation between subfoveal CT values using enhanced depth imaging (EDI) SD-OCT (Spectralis) and SS-OCT (Triton) (see Supplementary Table).

#### Choroidal Vessel Distribution and Vascularity Index

Choroidal vessel distribution (Haller's layer) was analyzed using en face OCT images extracted from the 9 × 9-mm OCT-A scans using a Bruch's membrane–CSI slab that covered the entire choroidal thickness (Figs. 1C, 1D). Because one of the main features of CSC is the presence of large choroidal vessels passing through the fovea, we wanted to determine if this condition was also present in healthy eyes. The choroidal vessel distribution was considered symmetrical when the vessels were equally distributed on either side of a horizontal line passing through the macula.

**Figure 2.**
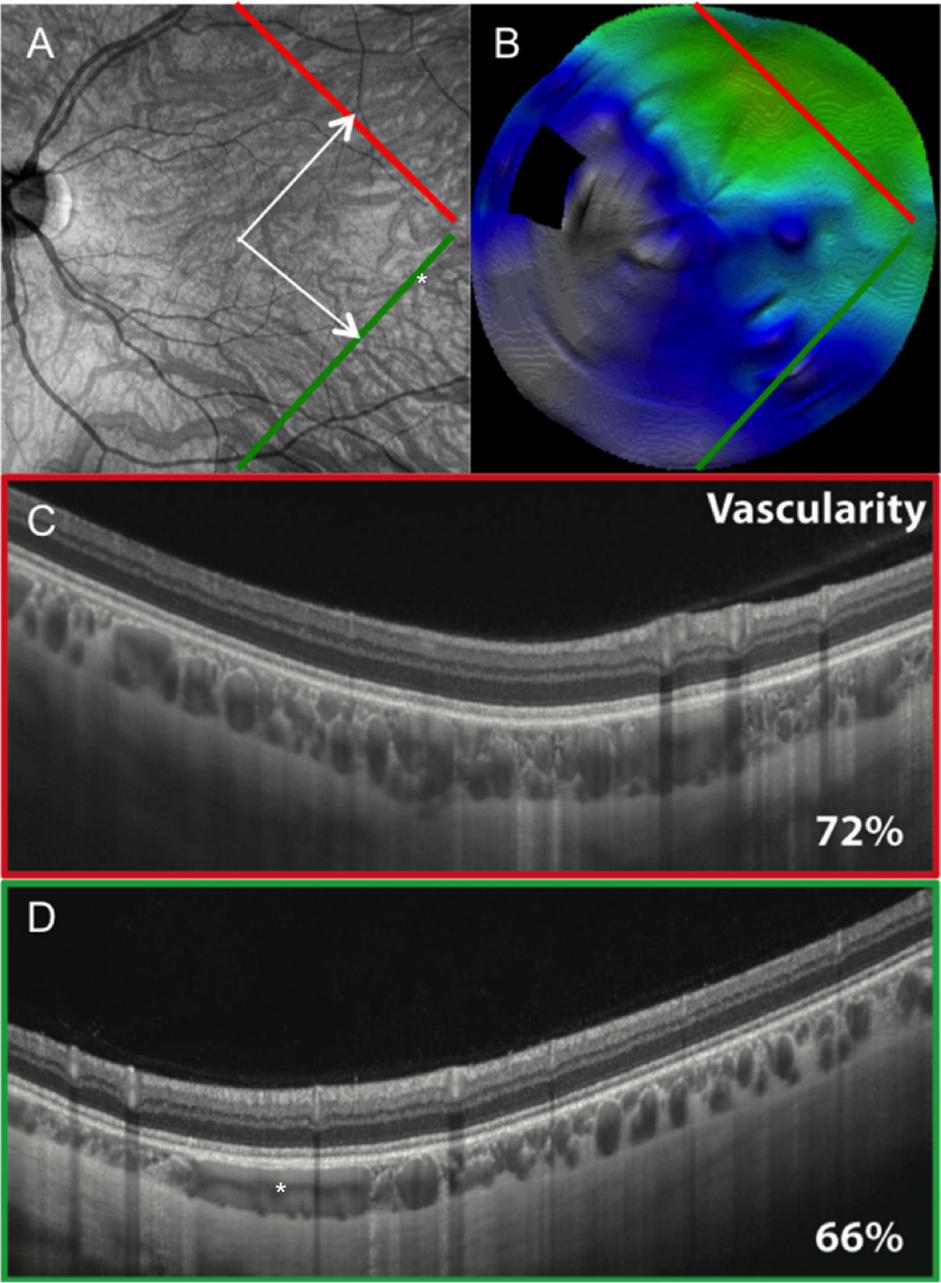
Vascularity index measurement. The vascularity index (VI) corresponds to the surface of the vessel lumen divided by the overall surface of the choroid. Two B-scans passing perpendicularly to the direction of the most choroidal veins in asymmetrical patterns were acquired superiorly and inferiorly with respect to the horizontal axis, at equidistance from the fovea, in the temporal region. The figure shows (**A**) a 9 × 9-mm en face OCT-A image and (**B**) the corresponding 12-mm radial CT map of a medium-thin asymmetrical choroid. The area of vessel lumen (dark on OCT B-scans) was determined based on a semiquantitative method. (**C**) B-scan used for VI measurement in the superotemporal region. (**D**) B-scan used for VI measurement in the inferotemporal region. In this example, the VIs were 72% and 66%, respectively, in the thickest and thinnest area of the CT map. In this image, one vessel is tangential to the B-scan, visible on the en face image (asterisk).

 The choroidal vascularity index (VI) is the ratio of luminal area to total choroidal area (from Bruch's membrane to the CSI). To better visualize the vessel lumen, two B-scans passing perpendicularly to the direction of the choroidal veins were acquired superiorly and inferiorly with respect to the horizontal axis, at equidistance from the fovea, in the temporal region (Fig. 2). This measurement better represents CT asymmetry between the superior and inferior choroid than a single horizontal measurement. The area of vessel lumen (dark on OCT scans) was determined on B-scans based on a semiquantitative method. Briefly, B-scans were exported as high-quality TIFF images and binarized for dark and light objects using MetaMorph Premier software (Molecular Devices, San Jose, CA, USA). The accuracy of the binarization was verified after manual delineation of dark areas. This analysis was not planned initially and was added after study initiation.

### Statistical Analysis

Statistical analysis was performed using GraphPad Prism 6 software (GraphPad Software, La Jolla, CA, USA). Quantitative variables were described using mean and SD values, and categorical variables were described using absolute and relative frequencies. One-way ANOVA and post hoc multiple comparisons were used to compare the CT in each single macular subfield. Paired *t*-tests and chi-squared and Fisher's exact tests were used when applicable. Correlations between continuous quantitative variables were performed by calculating the Spearman correlation coefficients (*r*). The level of significance was set at *P* < 0.05.

## Results

### Subject Characteristics

Sixty-six eyes of 33 Caucasian subjects were screened, and 64 eyes were included (Table 1). Two eyes were excluded due to poor image quality. In this cohort, the sex ratio was 1. The mean age was 37 years (range, 23–55 years; SD = 10). The mean SE was –0.7 D (range, –4.2 to +1.9; SD = 1.4), and the mean AL was 24 mm (range, 22–26; SD = 0.9). Visual acuity was 20/20 in all examined eyes, and the mean IOP was 13.1 mm Hg (range, 7–20; SD = 2.9). All subjects had normal blood pressure at the time of the evaluation, and there was no correlation between the choroidal thickness and the systolic or diastolic blood pressure (all *P* > 0.05).

**Table 1.** Subject Demographics and Clinical Characteristics (N = 64 Eyes)

Demographic	Value	Range (SD)
Age (y), mean (median)	37.0 (34.2)	23 to 55 (10.0)
Gender (female/male), n (%)	31 (48.5)/33 (51.5)	—
Systolic blood pressure (mm Hg), mean (median)	128.0 (128.0)	100 to 140 (14.0)
Diastolic blood pressure (mm Hg), mean (median)	70.6 (71.0)	50 to 88 (9.3)
Spherical equivalent (D), mean (median)	–0.7 (–0.6)	–4.2 to 1.9 (1.4)
Sphere (D), mean (median)	–0.3 (–0.5)	–4.0 to 3.0 (1.4)
Cylinder (D), mean (median)	–0.7 (–0.5)	0.0 to –3.0 (0.6)
Axial length (mm), mean (median)	24.0 (24.1)	22 to 26 (0.9)
Anterior chamber depth (mm), mean (median)	3.5 (3.5)	2.7 to 4.1 (0.3)
Intraocular pressure (mm Hg), mean (median)	13.1 (13.0)	7 to 20 (2.9)

### Quantitative Analysis of Choroidal Thickness

**Figure 3.**
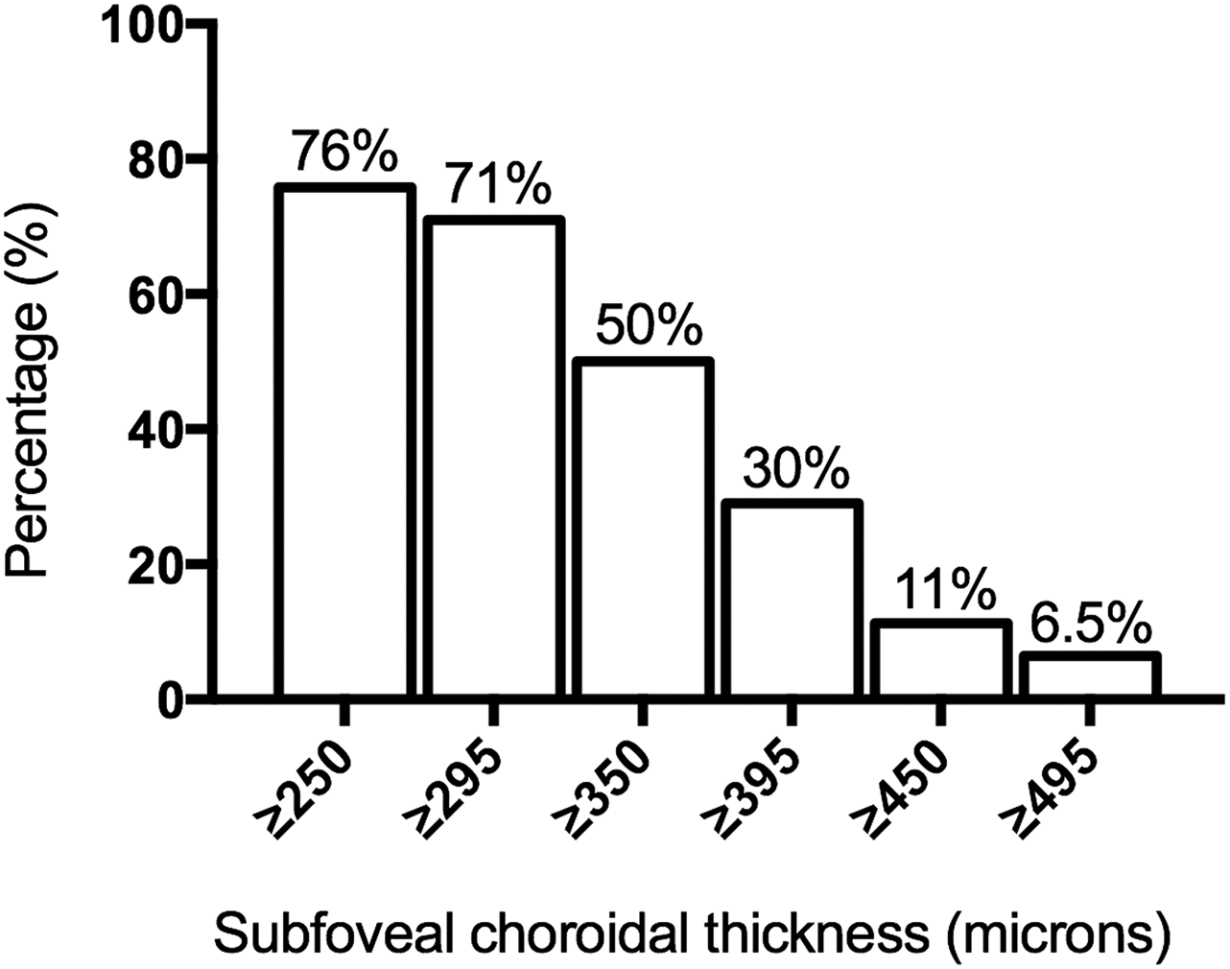
Graphical representation of subfoveal choroidal thickness distribution according to different thickness thresholds. The *x*-axis is the distribution of eyes according to subfoveal CT thresholds; the *y*-axis is the percentage of eyes. More than 50% of eyes had a subfoveal CT greater than 350 µm. Subfoveal CT measurements (central 1 mm) were performed using a Triton swept-source OCT.

Manual correction on the 12-mm radial scans was needed in 80% of cases for the CSI and in 10% of cases for the inner margin of the choroid (Bruch's membrane/RPE complex) (Table 2). CT values in each macular subfield of the ETDRS map are shown in Table 2, and the distribution of subfoveal CTs (central 1 mm) according to various thresholds is presented in Figure 3. Briefly, the mean subfoveal CT (mean CT in the central 1 mm) was 340 ± 99 µm and was >395 µm in 30% of cases. The mean total macular CT (mean CT of all nine choroidal subfields of the ETDRS grid) was 316 ± 95.1 µm. The mean CT was greater than 300 µm in all locations of the ETDRS map except in the NOM (mean CT in the NOM, 253 µm; range, 82–487 µm; SD = 94.1). Statistical analysis showed that the CT at the NOM was significantly thinner than in the other subfields of the ETDRS map (1-way ANOVA; *P* < 0.001). The temporal and superior fields were thicker than the nasal and inferior ones, respectively, in 92.6% and 72% of cases. CT values in the studied subfields were not independent; the subfoveal CT was representative of the overall choroidal macular thickness (total macula: *r* = 0.98, *P* < 0.0001) including the NOM (*r* = 0.9, *P* < 0.0001). There was a strong correlation among all CT values and age, AL, and SE (*r* < –0.4, *P* < 0.05; *r* > 0.7, *P* < 0.0001; and *r* > 0.6, *P* < 0.001, respectively), and there was no correlation between the choroidal thicknesses and the systolic or diastolic blood pressure (all *P* > 0.05). CT values showed a strong symmetry between both eyes in all locations (all *r* > 0.9, *P* < 0.0001).

**Table 2.** Topographic Variations of Choroidal Thickness Based on 12-mm SS-OCT Scans

	Mean (µm)	Range (SD)	CV
SCT	340.0	154–584 (99.0)	0.30
Inner ring (3-mm radius)			
TIM	338.0	144–564 (95.5)	0.30
NIM	308.0	125–550 (101.8)	0.33
SIM	346.0	119–566 (91.5)	0.28
IIM	335.0	106–584 (103.5)	0.33
Outer ring (6-mm radius)			
TOM	322.0	148–508 (87.0)	0.29
NOM	253.0^*^	82–487 (94.1)	0.37
SOM	337.5	117–529 (88.2)	0.27
IOM	315.0	100–533 (98.0)	0.33
Total macula	316.0	115–544 (95.1)	0.30
Thickest choroidal point	395.0	223–548 (87.2)	—

All measurements were based on the 12-mm scans. The total macula represents the mean CT of all nine subfields. CT, choroidal thickness; SCT, subfoveal CT in the central 1 mm; TIM, temporal inner macula; NIM, nasal inner macula; SIM, superior inner macula; IIM, inferior inner macula; TOM, temporal outer macula; NOM, nasal outer macula; SOM, superior outer macula; IOM, inferior outer macula (IOM); CV, coefficient of variation; SD, standard deviation.

^*^One-way ANOVA versus SOM, TOM, and IOM (*P* < 0.001).

### Location of the Thickest Choroidal Point

**Figure 4.**
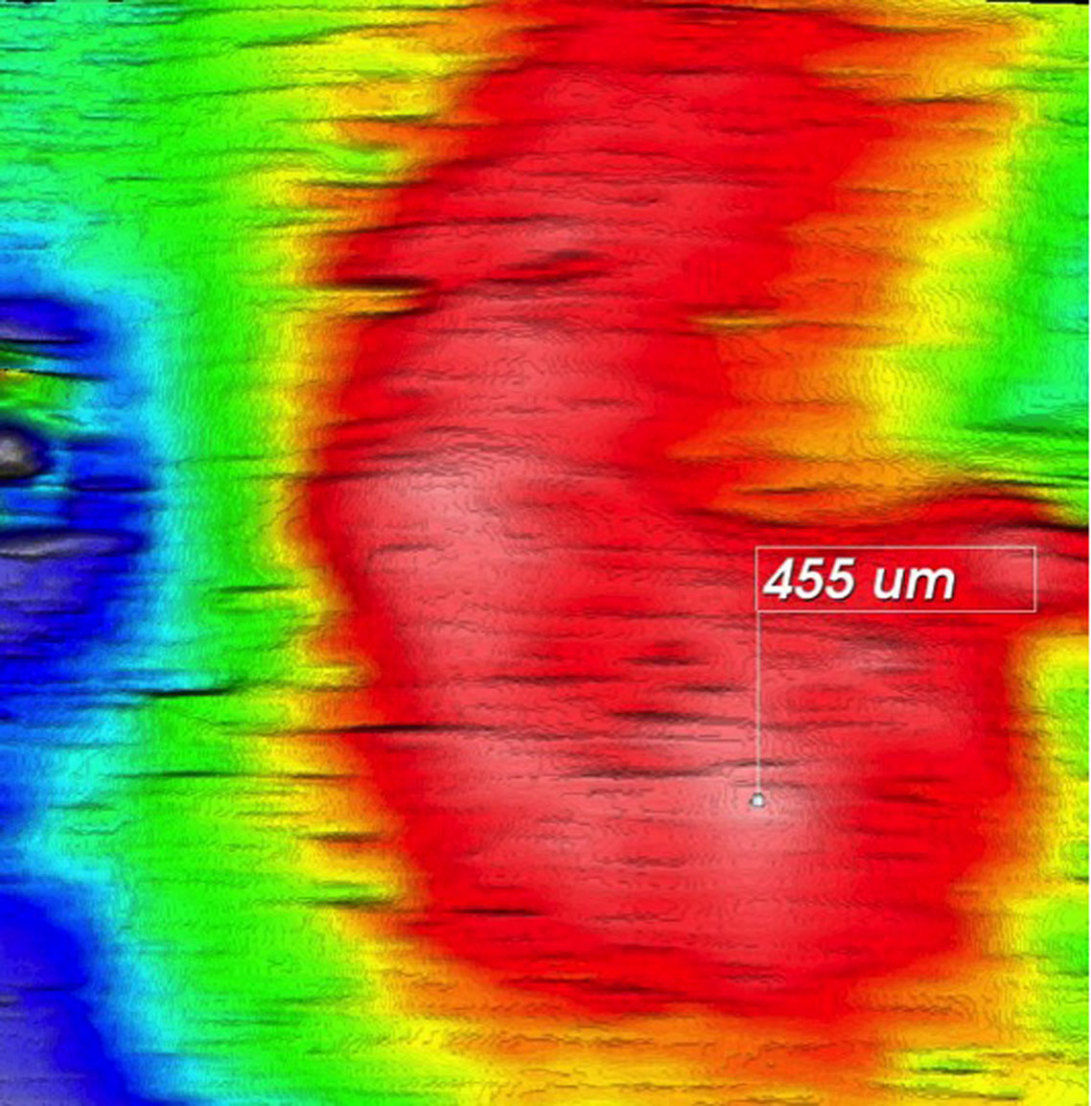
Determination of the thickest choroidal point. The thickest choroidal point (in this example, 455 µm) was obtained from the measurements made on the 9 × 9-mm OCT map based on the built-in manufacturer's software (Topcon DRI OCT Triton).

The mean CT at the TCP was 395 µm (range, 223–548; SD = 92.4) and was strongly correlated between both eyes (*r* = 0.9 between the right and left TCP; *P* < 0.0001) (Table 3, Fig. 4). The TCP was subfoveal in 1.8% of cases. Regardless of the CT, the TCP was most often located in the superior (72.2%) and temporal hemifields (72.2%) and mainly beyond the 3-mm ring of the ETDRS map (77.6%) (Table 3). The proportion of inferiorly located TCPs was higher in asymmetrical patterns (42.1%) than in symmetrical ones (21.9%) (*P* = 0.002). The TCP was located where the choroid had the highest choroidal vein density (as seen on en face OCT-A scans) in all cases.

**Table 3.** Location of the Thickest Choroidal Point

TCP	OD, n (%)	OS, n (%)	Total, n (%)
Subfoveal (<1 mm)	1 (3.6)	0 (0.0)	1 (1.8)
1–3 mm	5 (17.8)	7 (27.0)	12 (22.2)
3–6 mm	15 (53.6)	7 (27.0)	22 (40.7)
>6 mm	7 (25.0)	12 (46.0)	19 (35.2)
Superiorly	20 (71.4)	19 (73.0)	39 (72.2)
Inferiorly	8 (28.6)	7 (27.0)	15 (27.8)
Temporally	20 (71.4)	19 (73.0)	39 (72.2)
Nasally	8 (28.6)	7 (27.0)	15 (27.8)

The millimetric values are diameters (ETDRS map). OD, right eye; OS, left eye; TCP, thickest choroidal point.

### Qualitative Analysis of Choroidal Thickness Maps

With regard to thickness patterns, 38.7% of choroids were classified as thick (i.e., at least 50% of the 12 × 12-mm map surface > 395 µm), whereas 61.3% were considered as medium or thin. On average, 60.4% of the CT map surface was thicker than 395 µm (range, 50%–81%; SD = 9.5) in thick choroids. The mean subfoveal CTs of thick and medium-thin choroids were, respectively, 429.2 µm (range, 352–584; SD = 61.1) and 275 µm (range, 135–361; SD = 73), and the mean total macular CTs were, respectively, 405.4 µm (range, 337–543; SD = 55) and 261 µm (range, 115–365.5; SD = 69.2).

**Figure 5.**
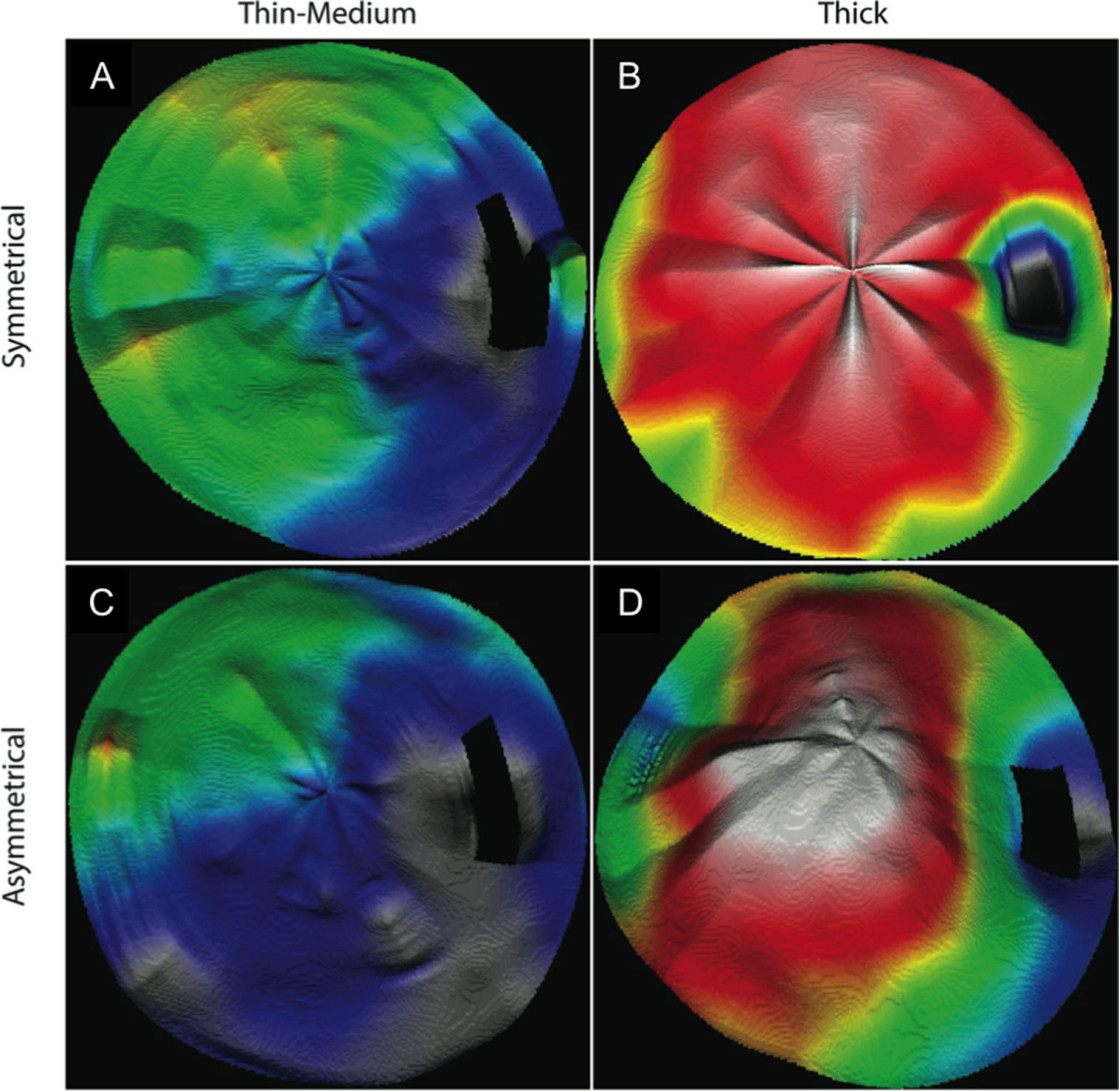
Choroidal thickness maps obtained from 12-mm radial scans in thin and thick choroids, with symmetrical or asymmetrical patterns with respect to the horizontal axis. (**A**) Symmetrical pattern with respect to the horizontal axis in a medium-thin choroid. (**B**) Symmetrical pattern in a thick choroid. (**C**) Asymmetrical pattern in a medium-thin choroid. (**D**) Asymmetrical pattern in a thick choroid. Color code: red, >400 µm; yellow, 300–400 µm; green, 200–300 µm; blue, <200 µm.

For top-to-bottom symmetry, the CT pattern was symmetrical along a horizontal axis passing through the fovea in 58% of cases (Figs. 5A, 5B) and asymmetrical in 42% of cases (Figs. 5C, 5D). The distribution of symmetrical and asymmetrical CT patterns was similar in thick and medium-thin choroids (58.3% vs. 41.7% and 57.9% vs. 42.1%, respectively; *P* = 0.95). In the whole cohort, the asymmetry of CT maps was oriented inferotemporally or superotemporally in half of the cases; however, it was oriented inferotemporally in 89.9% of thick choroids and superotemporally in 75% of medium-thin choroids (*P* < 0.0001).

No statistical difference was found in terms of age, gender, sphere, cylinder, SE, IOP, or AL between eyes with symmetrical and asymmetrical CT maps or between eyes with superiorly or inferiorly skewed CT patterns (all *P* > 0.05).

### Relationship between the Choroidal Thickness Pattern and the Choroidal Vessel Orientation/Layout

**Figure 6.**
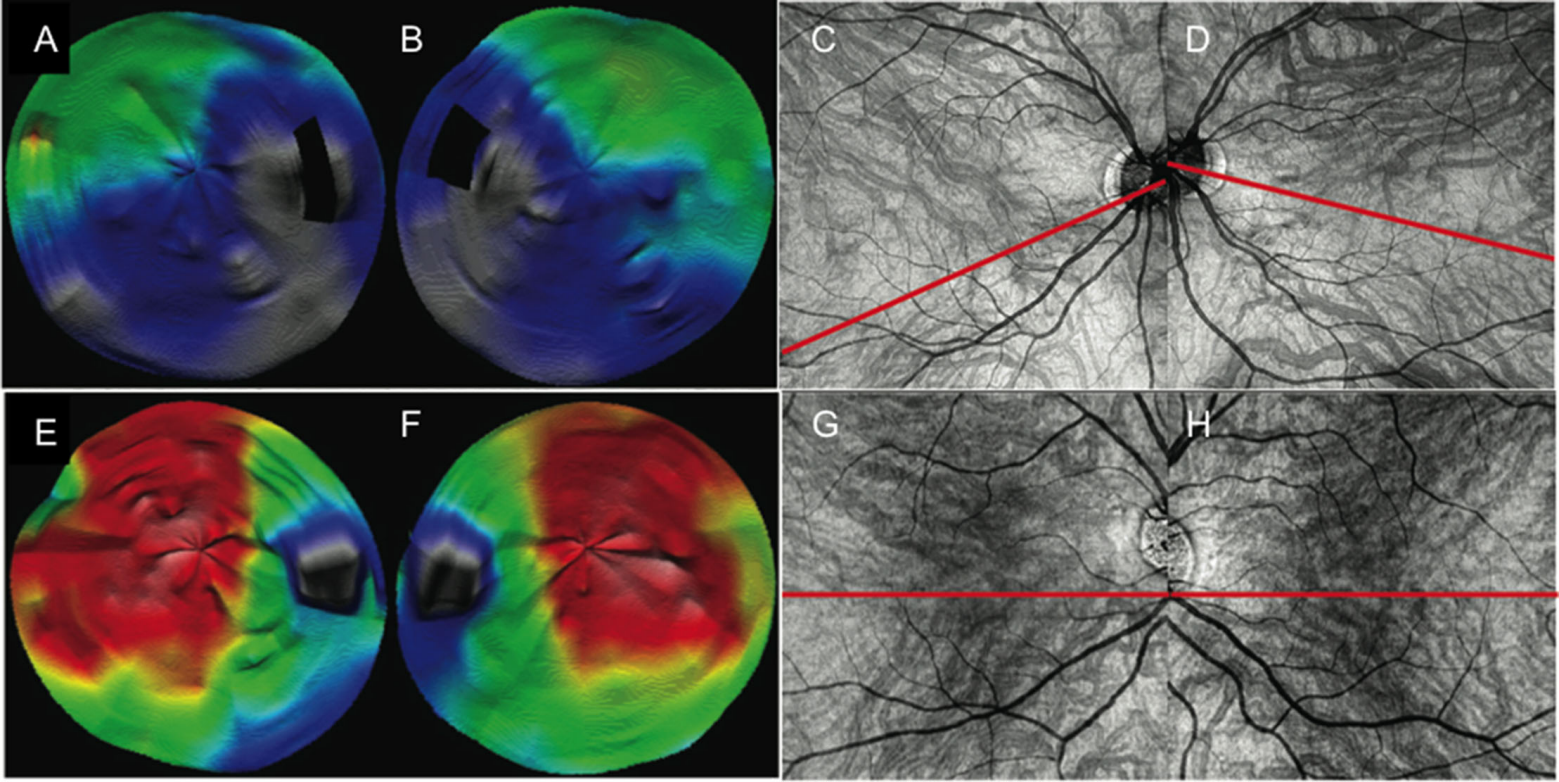
Correspondence between choroidal thickness maps and topography of choroidal veins on en face OCTA images. Choroidal thickness (CT) maps obtained from 12-mm radial scans: (**A**, **B**) medium-thin choroids and (**E**, **F**) thick choroids); corresponding en face OCT-A images of the large choroidal vessels: (**C**, **D**) medium-thin choroids and (**G**, **H**) thick choroids. Asymmetrical CT patterns (**A**, **B**) are characterized by an asymmetrical distribution of the choroidal veins, with the presence of oblique watershed zones with respect to the horizontal axis (**C**, **D**). Symmetrical CT patterns (**E**, **F**) correspond to a symmetrical distribution of these veins with respect to the horizontal axis (**G**, **H**). Color code: red, >400 µm; yellow, 300–400 µm; green, 200-300 µm; blue, <200 µm.

The asymmetrical patterns corresponded to an asymmetrical distribution of the choroidal veins showing an oblique watershed zone (Figs. 6A–6D). Conversely, the symmetrical patterns corresponded to a symmetrical distribution of these veins, with a watershed zone on the horizontal axis (Figs. 6E–6H). When taking into account the whole cohort, the watershed zone between the superior and inferior temporal choroidal veins was horizontal passing through the macula in 63.8% of cases. It was oblique superiorly in 18.9% of cases (i.e., thicker inferior choroid) and oblique inferiorly in 17.3% of cases (i.e., thicker superior choroid). This distribution corresponded to that of symmetrical versus asymmetrical CT patterns described earlier.

### Choroidal Vascularity

The mean VI was calculated in eight eyes with asymmetrical CT patterns, for which additional scans, perpendicular to the direction of choroidal veins, were available. The mean VI values were 75.6% and 74%, respectively, in the thickest and thinnest areas of CT maps (*P* = 0.78). The CT value appeared to depend mainly on vessel lumen (VI > 50%). The VI remained stable regardless of the CT variations in thick and thin areas in a given eye (Fig. 2).

### Pachyvessels and Autofluorescence

**Figure 7.**
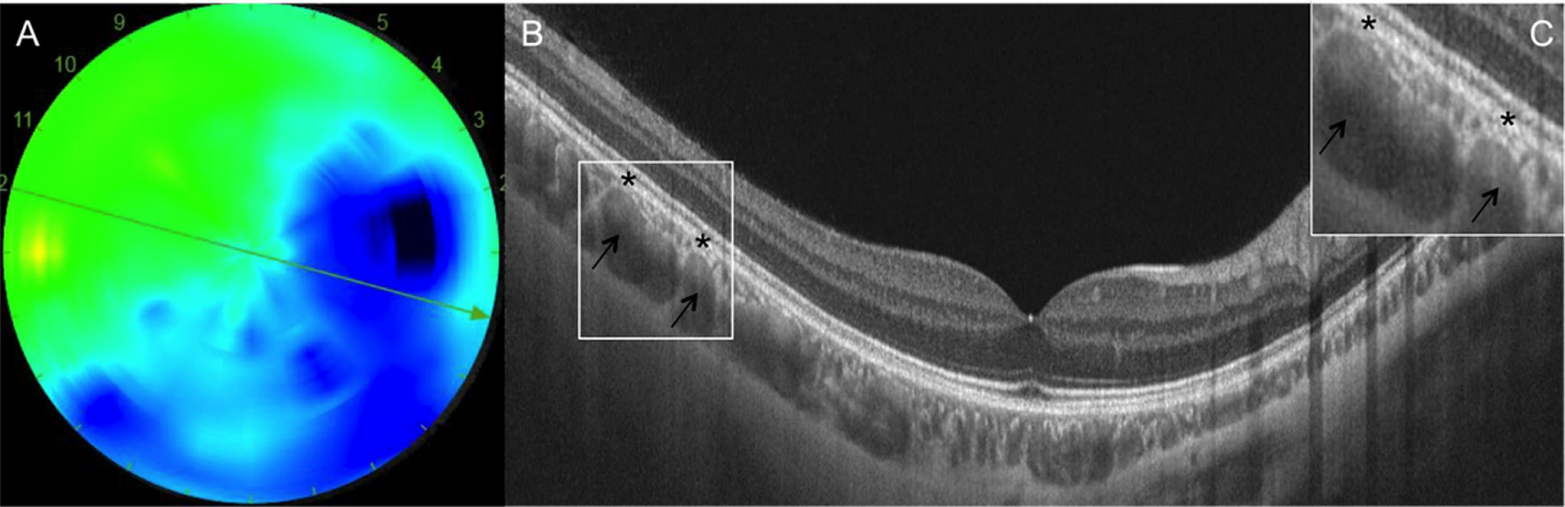
Example of large choroidal vessels in a medium-thin choroid. (**A**) The 12-mm CT map shows an asymmetrical CT pattern. Color code: red, >400 µm; yellow, 300–400 µm; green, 200–300 µm; blue, <200 µm. (**B**) The OCT B-scan corresponding to the green arrow in (**A**) shows the presence of large choroidal vessels, presumably veins (black arrows), occupying almost the entire CT, with no obvious choriocapillaris thinning (black asterisks). (**C**) Insert showing the magnification of the area of interest, the white square in (**B**); the choriocapillaris is still visible between the large vessel walls and the retinal pigment epithelium (black asterisks).

Large choroidal vessels occupying most of the CT were found in 44.4% of eyes, including eyes with thick and medium-thin choroids (Fig. 7). However, in no case was the overlying choriocapillaris totally compressed by the large vessels, which were never in direct contact with the Bruch's membrane. Ultra-wide-field autofluorescence imaging did not reveal any RPE abnormalities in this cohort.

### Subgroup Analysis

To further limit the effect of age, analyses were performed in the subpopulation of subjects < 40 years of age (subgroup 1). Forty-nine eyes of 25 subjects were included. The mean age was 33.1 years (range, 23–40 years; SD = 6.6). The mean SE was 0.6 D (range, –4.2 to 1.9 D; SD = 1.4). The mean AL was 24.1 mm (range, 22–26 mm; SD = 1.0). The quantitative analysis of CT in the different locations of the ETDRS map is shown in Table 4. Briefly, the same trends were observed in this subgroup analysis, as the nasal area was the thinnest, and the temporal and superior areas were the thickest, especially in the outermost zones of the ETDRS map. Forty-nine percent of choroids were classified as thick and 51% as medium-thin. The mean subfoveal CT was 349 ± 89.2 µm, and it was >395 µm in 32% of cases, as in the whole cohort. Qualitatively, the symmetric patterns accounted for 53.2% of cases, and among the asymmetrical patterns 59% showed an inferiorly oriented thickness profile. The symmetry of thickness patterns depended on the distribution of large vessel density. For the subgroup of subjects > 40 years of age (subgroup 2), the same trends were observed. The mean age was 51 years (range, 45–55 years; SD = 6.8). The mean SE was –0.8 D (range, –3 to 1.5 D; SD = 1.4), and the mean AL was 23.8 mm (range, 22.8–24.0 mm; SD = 0.7). The mean subfoveal CT was 315.3 ± 125 µm, and it was >395 µm in 20% of cases, as for subgroup 1 (*P* = 0.52). Qualitatively, the symmetric patterns accounted for 53.3% of cases (*P* = 1 vs. subgroup 1). Among the asymmetrical patterns, 100% showed a superiorly oriented thickness profile (*P* = 5.02e-15 vs. subgroup 1). The symmetry of thickness patterns depended on the distribution of large vessel density.

**Table 4.** Topographic Variations of Choroidal Thickness Based on 12-mm SS-OCT Scans for the Subgroup of Subjects <40 Years of Age (N = 47)

		Mean (µm)	Range (SD)
SCT		349.0	154–503 (89.2)
Inner ring (3-mm radius)			
	TIM	347.0	144–527 (88.6)
	NIM	322.0	125–472 (90.4)
	SIM	352.0	119–506 (83.8)
	IIM	347.0	106–511 (93.3)
Outer ring (6-mm radius)			
	TOM	334.0	148–504 (80.3)
	NOM	262.0^*^	82–411 (83.4)
	SOM	344.0	117–487 (78.7)
	IOM	329.0	100–488 (86.5)

All measurements were based on the 12-mm scans. The total macula represents the mean CT of all nine subfields. CT, choroidal thickness; SCT, subfoveal CT in the central 1 mm; TIM, temporal inner macula; NIM, nasal inner macula; SIM, superior inner macula; IIM, inferior inner macula; TOM, temporal outer macula; NOM, nasal outer macula; SOM, superior outer macula; IOM, inferior outer macula (IOM); CV, coefficient of variation; SD, standard deviation.

^*^One-way ANOVA versus SOM, TOM, and IOM (*P* < 0.05).

## Discussion

The choroid plays an important role in the pathophysiology of various diseases such as age-related macular degeneration, CSC, pathologic myopia, uveitis, and neoplastic disorders. In this context, characterizing normal choroidal features, and more particularly the CT, has raised growing interest, especially during the last decade, which has seen the advent of the pachychoroid concept. The definition of pachychoroid^11^ has evolved over time to be based not only on the CT but also on a qualitative description associating various criteria using en face SS-OCT,^46^ including (1) the CT, (2) pathologically dilated veins in the Haller's layer (pachy-veins), and (3) a thinning of Sattler's and choriocapillaris layers, the thresholds of which remain to be defined in a standardized reproducible way. In fact, many healthy, normal subjects have thick choroids in the absence of pathology; therefore, the definition of pachychoroid includes more than only the choroidal thickness. The cut-off between a normal and thickened (i.e., pathological) choroid in the same age group and emmetropic eyes has not been clearly determined, although a subfoveal threshold of 395 µm has recently been proposed.^9^^,^^12^ The aim of this study was to describe and analyze both quantitatively and qualitatively the features of the normal choroid in order to provide reference criteria to better identify pathological conditions involving the choroid, such as CSC in which the choroid is thickened.

The commercially available SS-OCT/OCT-A device that was used here provides in a single acquisition a CT map based on 12 12-mm radial B-scans and a 9 × 9-mm thickness and vascular map based on dense parallel B-scans, which allows a direct point-to-point correlation of all features of interest. In this series of healthy subjects ages 25 to 55 years with ALs in the normal range, we found a mean subfoveal CT of 340 ± 99 µm, which is within the limits of recent publications that used SS-OCT and found that the subfoveal CT varied from 229 to 379 µm.^13^^,^^20^^,^^26^^,^^27^^,^^38^^,^^39^^,^^47^^–^^51^ The significant variation in subfoveal CT values could be explained by differences in patient demographics (mainly the age range^13^^,^^14^^,^^16^^,^^45^ and the refractive error or AL^5^^,^^45^^,^^52^) and the way in which CT was measured (subfoveal or within the central 1-mm diameter, swept-source or EDI SD-OCT). In our series, the subfoveal CT was >395 µm in 30% of cases, a significant proportion which has not been highlighted before. This finding means that the definition of pachychoroid could not only rely on a choroidal thickening, as recently reported by Cheung et al.^12^ We also investigated the distribution of the subfoveal CT in healthy eyes of patients younger than 55 years and showed that 50% of them had a subfoveal CT > 350 µm (Fig. 3). We then used another definition of a thick choroid, which was a CT > 395 µm in more than 50% of the 12 × 12-mm CT map surface, the threshold of 395 µm being chosen because it was the mean TCP value. According to this definition, the choroid was thick in 38.7% of cases, but medium-thin was the most common choroidal pattern (61.3%).

Beyond the foveal area, the CT was thicker superiorly and temporally in most cases, as previously shown by others,^28^^,^^33^^,^^53^ but in 27.8% of cases it was located inferiorly, a finding that is not usually reported,^21^ except in hyperopic eyes.^20^ Of note, the TCP was not subfoveal in 98.2% of cases, and, on average, it was 395 µm thick with large inter-individual variations (range, 223–548 µm). The TCP was most often located in the superior (72.2%) and temporal (72.2%) hemifields and mainly beyond a 3-mm diameter from the fovea (76%), regardless of the CT.

Pachyvessels were found in 44.4% of eyes, but, even when the dilated vessels occupied almost all the CT and even in eyes with thick choroids, the choriocapillaris was always distinct above these large vessels (Fig. 7). The fact that >30% of our healthy subjects 55 years of age or less showed thick choroids emphasized the importance of not defining the pachychoroid feature based only on CT values.^12^

The VI remained the same (about 70%) in the thinnest and thickest parts of asymmetrical choroids. It has also been shown that the vascularity of the subfoveal choroid was independent of its thickness. Therefore, although the distribution of the large outer choroidal vessels seemed to play a major role in determining the global CT pattern, our results also suggest that the thickness of both the vascular lumen and the stroma are concurrently increased in the thickest choroids in the studied age group. This is in accordance with the recent study of Goud et al.,^56^ who showed that the CVI does not show any significant difference in different rings, subfields, or quadrants of the ETDRS map in the macular area. This important finding could be of interest for the interpretation of pathological features.^57^ The choroidal VI has been suggested to be a potential marker of pathology in uveitis, where it has been shown to be increased in the acute stages of MEWDS disease,^58^ and in CSC, where it has been suggested to be decreased in the chronic neovascularized forms of the disease.^59^

CT symmetry between the superior and inferior parts of the temporal choroid showed a large inter-individual variation. The CT was symmetrical with respect to the temporal raphe in 58% of cases, as observed by Mori et al.^54^ for the distribution of choroidal veins on indocyanine green angiography (ICGA) and by Hiroe and Kishi,^55^ who has found a symmetrical distribution of choroidal veins in 62% of cases based on ICGA and SS-OCT-A. In asymmetric cases, the thickest part of the choroid was more often superotemporal in medium-thin choroids and more often inferotemporal in thick choroids. The top-to-bottom arrangement of CT patterns appeared to correlate with the relative density of the large choroidal veins with respect to the horizontal axis as seen on en face OCT-A images.

Finally, we found that the choroidal pattern was symmetrical between both eyes, including the symmetry of CT values in all locations of the ETDRS map and the symmetry of the CT pattern and choroidal vessel arrangement with respect to the horizontal axis. CT symmetry in specific locations of the posterior pole has already been reported,^19^^,^^21^ and others have suggested a thicker macular nasal choroid in right eyes compared to left eyes.^39^ On the other hand, data on the symmetry of the choroidal vessel pattern arrangement are limited.

Our study has some limitations. First, the sample size is small and it would be interesting to confirm these findings in larger cohorts. Second, we included subjects aged 55 years or less, who cannot be used for comparison with older age-related macular degeneration patients. It is also the case for patients with chronic CSC; although the disease is often diagnosed by the age of 40, many patients (especially those with neovascular CSC) are older. Third, the lateral scale of the OCT scans was not corrected according to the AL; however, due to the narrow range of the AL (22 mm < AL < 26 mm), changes were considered negligible (+5% to –9%). At most, the absence of correction tended to underestimate the CT in the cases with shorter AL, and the opposite was observed in the cases with longer AL. This did not affect the distribution of the CT in medium-thin or thick choroids. Fourth, we did not observe any correlation between the choroidal venous pattern observed on en face OCT-A and that observed on ultra-wide-field ICGA because of the invasive nature of this examination in healthy subjects. Finally, the 395-µm threshold that was used herein to define a thick choroid was chosen based on a recent publication that set this limit based on a spectral domain EDI technology.^9^ To overcome this rather arbitrary value, we presented the distribution of subfoveal CTs across a wide range of thresholds to provide a more comprehensive overview of the normal choroid in this healthy population.

## Conclusions

Wide-field SS-OCT coupled with OCT-A provides reliable data on choroidal thickness and topography. This study showed that there are different choroidal patterns in the normal population, including thick and thin choroids—symmetrical and asymmetrical patterns without any impact on vision. Thirty percent of eyes of healthy individuals 55 years of age and less had a subfoveal CT > 395 µm, and the maximum CT was usually outside the foveal center. Such variations were not associated with any RPE anomalies on autofluorescence, and the choriocapillaris was always visible, even in thick choroids. Choroidal vascularity index was stable across various choroidal thicknesses, and the choroidal pattern was symmetrical between the eyes of an individual.

These findings should be taken into account when interpreting choroidal findings in chorioretinal diseases, especially the findings about the pachychoroid spectrum. Ongoing studies will investigate the pachychoroid states in the same way to be able to compare the results with those obtained with this “normal” cohort.

## Supplementary Material

Supplement 1
