# Supplementary material for: Topographic Variations of Choroidal Thickness in Healthy Eyes on Swept-Source Optical Coherence Tomography
Source: Invest Ophthalmol Vis Sci. 2020 Mar 20;61(3):38. doi: 10.1167/iovs.61.3.38 (PMC7401446; doi:10.1167/iovs.61.3.38)
Supplement: Supplement 1 [file iovs-61-3-38_s001.pdf]

Supplementary table: Correlation between enhanced depth imaging spectral domain and swept source OCT subfoveal choroidal thicknesses			
	**A. Subfoveal CT in the central 1mm (Topcon)**	**B. Subfoveal CT (Topcon)**	**C. Subfoveal CT (Spectralis EDI)**
**Mean (microns)**	382.7	401.8	398.0
**Median (microns)**	380.5	404.0	410.0
**SD**	103.6	106.1	101.3
**Range (microns)**	194-566	203-600	201-563

CT: choroidal thickness, EDI: enhanced depth imaging, SD: Standard deviation. A. Mean subfoveal CT in the central 1 mm measured with a swept source OCT technology (derived from 12mm radial scans, Triton^{TM}, Topcon medical systems, Tokyo, Japan). In B (Swept source OCT, Triton^{TM}, Topcon medical systems, Tokyo, Japan) and C (Spectral domain OCT, EDI, Spectralis^{TM}, Heidelberg, Germany), subfoveal CT represents the measure of CT with the calliper tangientially under the fovea.
Spearman correlation coefficient of A-B: r=0,98, p<0,05; and B-C: r= 0,93, p<0,0001 show that all measures are highly correlated. One way Anova of A vs B vs C, p=0.87. N=16 eyes
